# Cognitive Impairment In Treatment-Naïve Bipolar II and Unipolar Depression

**DOI:** 10.1038/s41598-018-20295-3

**Published:** 2018-01-30

**Authors:** Arthur D. P. Mak, Domily T. Y. Lau, Alicia K. W. Chan, Suzanne H. W. So, Owen Leung, Sheila L. Y. Wong, Linda Lam, C. M. Leung, Sing Lee

**Affiliations:** 1 Department of Psychiatry, Faculty of Medicine, The Chinese University of Hong Kong, Hong Kong, China; 2 Department of Psychology, The Chinese University of Hong Kong, Hong Kong, China

## Abstract

Cognition dysfunction may reflect trait characteristics of bipolarity but cognitive effects of medications have confounded previous comparisons of cognitive function between bipolar II and unipolar depression, which are distinct clinical disorders with some overlaps. Therefore, we examined the executive function (WCST), attention, cognitive speed (TMT-A) and memory (CAVLT, WMS-Visual reproduction) of 20 treatment-naïve bipolar II patients (BPII), 35 treatment-naïve unipolar depressed (UD) patients, and 35 age/sex/education matched healthy controls. The subjects were young (aged 18–35), and had no history of psychosis or substance use, currently depressed and meeting either RDC criteria for Bipolar II Disorder or DSM-IV-TR criteria for Major Depressive Disorder. The patients were moderately depressed (MADRS) and anxious(HAM-A), on average within 3.44 years of illness onset. Sociodemographic data and IQ were similar between the groups. UD patients had significantly slower cognitive speed and cognitive flexibility (WCST perseverative error). BPII depressed patients showed relatively intact cognitive function. Verbal memory (CAVLT List A total) correlated with illness chronicity only in BPII depression, but not UD. In conclusion, young and treatment-naïve BPII depressed patients differed from unipolar depression by a relatively intact cognitive profile and a chronicity-cognitive correlation that suggested a stronger resemblance to Bipolar I Disorder than Unipolar Depression.

## Introduction

Controversy exists as to the extent cognitive impairment found in Bipolar Disorders^1^ reflect trait characteristics for bipolarity^2–4^ or indicate neuroprogressive consequences of chronicity and episodic recurrence^3,5^. This is partly because of the methodological difficulty of recruiting drug-naïve patients. Yet, since both mood stabilisers and antipsychotic drugs, commonly used in the long-term treatment of bipolar disorders, have varied effects on cognition^6^, recruiting drug-naïve and ideally younger patients is essential for clarifying the inconclusive findings.

Another way to elucidate the role of cognitive impairment in bipolar disorders is to examine such impairment in bipolar II (BPII) depression vs unipolar depression (UD). BPII is the commonest bipolar subtype, but where diagnostic controversy frequently exists owing to its clinical features which often overlap with unipolar depression. The illness courses in both of the disorders are predominated by depressive states^7^. Up to one-third of patients diagnosed with UD would meet criteria for BPII^8^, and one-fifth would have diagnostic conversion to BPII in 5 years^9,10^. Increased chronicity and recurrent depressive phases^7^, reverse vegetative symptoms, mood reactivity, as well as antidepressant non-response in bipolar II depression, on the other hand, suggest a different pathophysiological substrate than UD^11^. It is therefore noteworthy that, in contrast to studies of depressed and medicated BPII patients which found impaired sustained attention^12^ and executive function^13^, Taylor Tavares *et al*.^14^ found the cognitive function of currently unmedicated BPII depressed patients (n = 17) to be relatively intact compared to unipolar depressed patients (n = 22). Unlike BPII patients, the latter showed prominent deficits including spatial working memory, attentional shifting, and tendency to sample loss trials on a gamble test. The patients in this study had, however, received psychotropic treatment in the past, with an average of 15–18 years of illness. The different types of medications (antidepressants versus mood stabilisers) received by unipolar and bipolar patients may have different sustained impact on the cognitive function studied. Common reasons for cessation of treatment in patients with bipolar disorders (e.g. impaired insight, secondary non-compliance when mental state was poor, or a relatively benign illness course rendering patients less committed to the need for continued psychopharmacological maintenance) may also have influenced the cognitive functions measured. With the long past illness histories and previous pharmacotherapy in these patients, it would also be more difficult to attribute results from the study to inherent endophenotypic differences between bipolar and unipolar depression.

Published data on cognitive function in BP-II depressed patients, apart from Tavares Taylor *et al*., are rare. We therefore set out to examine the cognitive function of young and treatment-naïve bipolar II and unipolar depressive patients with no history of psychosis or substance use, versus age/sex-matched controls to better elucidate trait-related variation in cognitive function in bipolar II disorder. They were then compared with age/sex-matched healthy controls. Our objectives were:

1. To examine the nature and differences of cognitive impairment in drug-naïve and young BPII depressed and unipolar depressed patients
2. To explore in BPII depressed and unipolar depressed patients the correlation between cognitive impairment with illness chronicity and current affective symptoms

## Results

In the years 2014–2017, 20 treatment-naïve and currently depressed subjects with Bipolar II Disorder (BPII), 35 treatment-naïve and currently depressed subjects with Major Depressive Disorder (UD) and 35 healthy controls (HC) were systematically recruited. The BPII, UD and HC groups were comparable in age, gender and other socio-demographic variables including occupational status, marital status and family income. **(**Table 1**)**.

**Table 1:** Sociodemographic data of participants.

	Healthy control (*n* = 35)	Unipolar depression (*n* = 35)		Bipolar II depression (*n* = 20)	X^2^	F	P
Gender, female: *n* (%)	23 (65.7)	20 (57.1)		16 (80)	0.22		0.22
Age, years: mean (s.d.)	22.95 (3.20)	24.94 (4.37)		23.20 (4.06)		2.67	0.08
Marital status, *n* (%)							
Married or cohabiting Separated abode Single	1 (2.9) 0 34 (97.1)		7 (20) 1 (2.9) 27 (77.1)	2 (10) 0 18 |(90)	7.04		0.13
Family income, less than HKD 30,000 monthly: *n* (%)	20 (57.1)		26 (74.3)	12 (66.7)	2.29		0.32
Education, years: mean (s.d.)	15.4 (1.22)		13.81 (1.82)	14.8 (2.04)		1.66	0.20
Unemployment, *n* (%)	1 (2.9)		7 (20)	4 (20)	5.44		0.07

### Clinical symptom and course

The BPII and UD subjects had MADRS and HAM-A scores of moderate severity, significantly higher than HC (ps < 0.001), but not significantly different between BPII and UD (ps > 0.05). Current average YMRS score in BPII subjects was low (4.30). UD and BPII subjects were comparable in the significantly reduced SF-36 physical and mental component summary scores compared to healthy controls (ps < 0.001), reflecting impaired health-related quality of life **(**Table 2**)**. BPII subjects had significantly earlier age of depressive onset than UD (p = 0.01), significantly longer time since first depressive onset (p = 0.01) and significantly more major depressive episodes than UD (p = 0.02). BPII subjects had on average 71.65 lifetime hypomanic episodes (six of the subjects reported more than monthly hypomanic episodes in at least 5 years of illness).

**Table 2:** Clinical symptoms and course of healthy control, unipolar depression and bipolar II depression.

	Healthy control (n = 35)	Unipolar depression (n = 35)	Bipolar II depression (n = 20)	F	p
Montgomery-Åsberg Depression Rating Scale, mean (s.d.)	0.15 (0.43)	23.20 (5.41)^a^	21.55 (9.67)^a^	164.45	<0.001**
Young Mania Rating Scale, mean (s.d.)	0.00 (0.00)	1.83 (2.82)^a,b^	4.30 (5.17)^a,b^	13.22	<0.001**
Hamilton Anxiety Rating Scale, mean (s.d.)	1.83 (2.82)	21.11 (8.80)^a^	19.50 (9.71)^a^	72.20	<0.001**
Age at onset of depression, mean (s.d.)		22.34 (4.98)^b^	18.30 (5.55)^b^	7.71	0.01*
Years since depression onset, mean (s.d.)		2.60 (2.78)^b^	4.90 (3.86)^b^	6.53	0.01*
Total number of major depressive episodes, mean (s.d.)		1.43 (0.70)^b^	2.60 (1.54)^b^	64.52	<0.001**
Total number of hypomanic episodes, mean (s.d.)			71.65 (106.5)		
Short-form 36 - physical component summary, mean (s.d.)	55.62 (4.06)	49.15 (7.52)^a**^	49.81 (7.01)^a**^	10.66	<0.001**
Short-form 36 - mental component summary, mean (s.d.)	54.04 (4.18)	27.90 (10.01)^a**^	26.96 (11.04)^a**^	103.44	<0.001**

**Correlation is significant at the 0.01 level (2-tailed); a: vs control p < 0.05; b: UD vs BD p < 0.05.

### Cognitive Variables

The average estimated IQ scores in the BPII, UD and HC groups were similar. **(**Table 3**)**. Analysis of variance (ANOVA) showed a main effect of HC/UD/BPII grouping on processing speed (TMT-A time to completion) (p < 0.001). Post-hoc Bonferroni correction showed significantly slower psychomotor speed (TMT-A completion) in UD compared to BPII (p = 0.001) and HC (p < 0.001) but indistinguishable between BPII subjects and HC. In a logistic regression model controlled for age, gender, years of education, current depression, mania and anxiety severity, lifetime number of depressive episodes and illness chronicity, TMT-A completion (p = 0.02, AOR = 0.85, 95%CI: 0.74–0.97) and lifetime number of depressive episodes (p = 0.02, AOR = 3.34, 95%CI: 0.84–1.06) significantly differentiated the BPII and UD diagnostic groups.

**Table 3:** Cognitive variables in Bipolar II depressed, Unipolar Depressed and Healthy subjects.

	HC (*n* = 35) Mean (sd)	UD (*n* = 35) Mean (sd)	BPII (*n* = 20) Mean (sd)	F	p		Cohen’s d	
						HC vs UD	HC vs BPII	UD vs BPII
**Frontal Executive Function**								
*Wisconsin card sorting test*								
%perseverative errors	7.03 (2.81)	10.09 (5.35)^a,^**	9.04 (3.95)	4.7	0.01**	0.72	0.59	0.22
Categories completed	5.89 (0.67)	5.6 (1.14)	5.8 (0.70)	0.93	0.4	0.31	0.13	0.21
**Attention and mental tracking**								
Digit Span-forward	10.11 (1.23)	9.94 (1.53)	9.99 (1.31)	0.22	0.8	0.12	0.1	0.04
Digit Span- Backward	7.43 (1.93)	7.06 (1.92)	7.55 (1.82)	0.54	0.59	0.19	0.13	0.26
Trail-making test A	24.75 (8.64)	31.79 (10.73)^a,**, b,**^	24.00 (5.00)^b,**^	7.23	0.001**	0.72	0.1	0.93
Trail-making test B	52.56 (21.62)	60.56 (20.34)	53.09 (15.13)	1.66	0.2	0.38	0.03	0.42
**Verbal fluency**								
Correct	34.09 (5.81)	32.37 (6.94)	32.45 (7.03)	0.71	0.5	0.34	0.25	0.01
Repetition	0.31 (0.68)	0.63 (1.14)	0.40 (0.60)	1.18	0.31	0.06	0.14	0.25
Intrusion	0 (0)	0.06 (0.24)	0 (0)	1.68	0.21	n/a	n/a	n/a
**Learning and memory**								
**Chinese Auditory Verbal Learning Test**								
List A Total Correct Response	53.69 (9.48)	52.14 (8.54)	56.75 (8.07)	1.74	0.18	0.17	0.35	0.15
Interference effect	0.97 (1.76)	0.74 (1.52)	0.95 (2.21)	0.12	0.88	0.14	0.01	0.19
Recognition	48.23 (2.41)	48.54 (1.84)	48.14 (4.08)	0.47	0.63	0.14	0.07	0.03
***Wechsler Memory Scale (visual reproduction)***								
Immediate recall	11.57 (2.54)	10.20 (2.61)	10.60 (2.84)	2.47	0.09	0.53	0.47	0.15
Delayed recall	10.89 (3.26)	9.47 (2.95)	10.50 (3.63)	1.68	0.19	0.46	0.11	0.31
Recognition	8.68 (3.61)	7.71 (3.50)	7.90 (3.13)	0.46	0.63	0.27	0.23	0.06

**Correlation is significant at the 0.01 level (2-tailed); a: vs control p < 0.05, bonferroni corrected; b: ud vs bd p < 0.05, bonferroni corrected.

On measures of frontal executive function, main group effects were found for WCST percentage of perseverative errors (p = 0.01). After Bonferroni correction, UD but not BPII subjects showed significantly increased percentage of perseverative errors compared to controls (p = 0.02) with a medium to large effect size (0.7).

### Cognitive correlates – Bipolar II Depressed Subjects

**(**Table 4**)** In BPII subjects, TMT-A completion and TMT-B completion both correlated with current anxiety. Current depressive and manic symptoms did not correlate with any cognitive parameters examined.

**Table 4:** Pearson correlation coefficients (Bootstrap BCa 95% confidence intervals) of correlations of cognitive tests (ES > 0.30 versus HC) with symptomatic and course variables in Bipolar II Depressed subjects.

Bipolar II Depressed subjects N = 20	MADRS	HAM-A	YMRS	Illness Chronicity	Number of hypomanic episodes	Number of depressive episodes		Cycling frequency
**Frontal Executive Function**								
WCST % perseverative error	−0.06(−0.38–0.31)	0.26 (−0.290.61)	−0.15 (−0.460.35)	−0.19 (−0.54–0.44)	0.01 (−0.30–0.58)	0.23 (−0.20–0.62)	−0.11 (−0.41–0.38)	
**Attention and mental tracking**								
TMT-A	−0.01 (−0.36–0.44)	0.62** (0.29–0.84)	−0.28 (−0.56–0.14)	−0.19 (−0.65–0.32)	−0.38 (−0.71–0.21)	−0.13 (−0.54–0.29)	−0.23 (−0.58–0.36)	
TMT-B	0.33 (−0.30–0.88)	0.72** (0.34–0.87)	−0.25 (−0.58–0.65)	−0.15 (−0.58–0.31)	−0.38 (−0.36–0.39)	−0.01 (−0.62–0.28)	−0.03 (−0.49–0.35)	
**Learning and memory**								
CAVLT- List A Total Correct Response	0.08 (−0.44–0.47)	0.21 (−0.20–0.64)	0.04 (−0.36–0.39)	0.46* (0.03–0.74)	0.10 (−0.23–0.39)	−0.10 (−0.53–0.27)	0.06 (−0.34–0.42)	
Wechsler Memory Scale (Visual Reproduction) Immediate recall	0.19 (−0.34–0.56)	−0.35 (−0.75–0.33)	−0.17 (−0.63–0.19]	−0.01 (−0.45–0.34)	0.13 (−0.41–0.49)	0.15 (−0.30–0.47)	0.32 (−0.02–0.59)	

**Figure 1:**
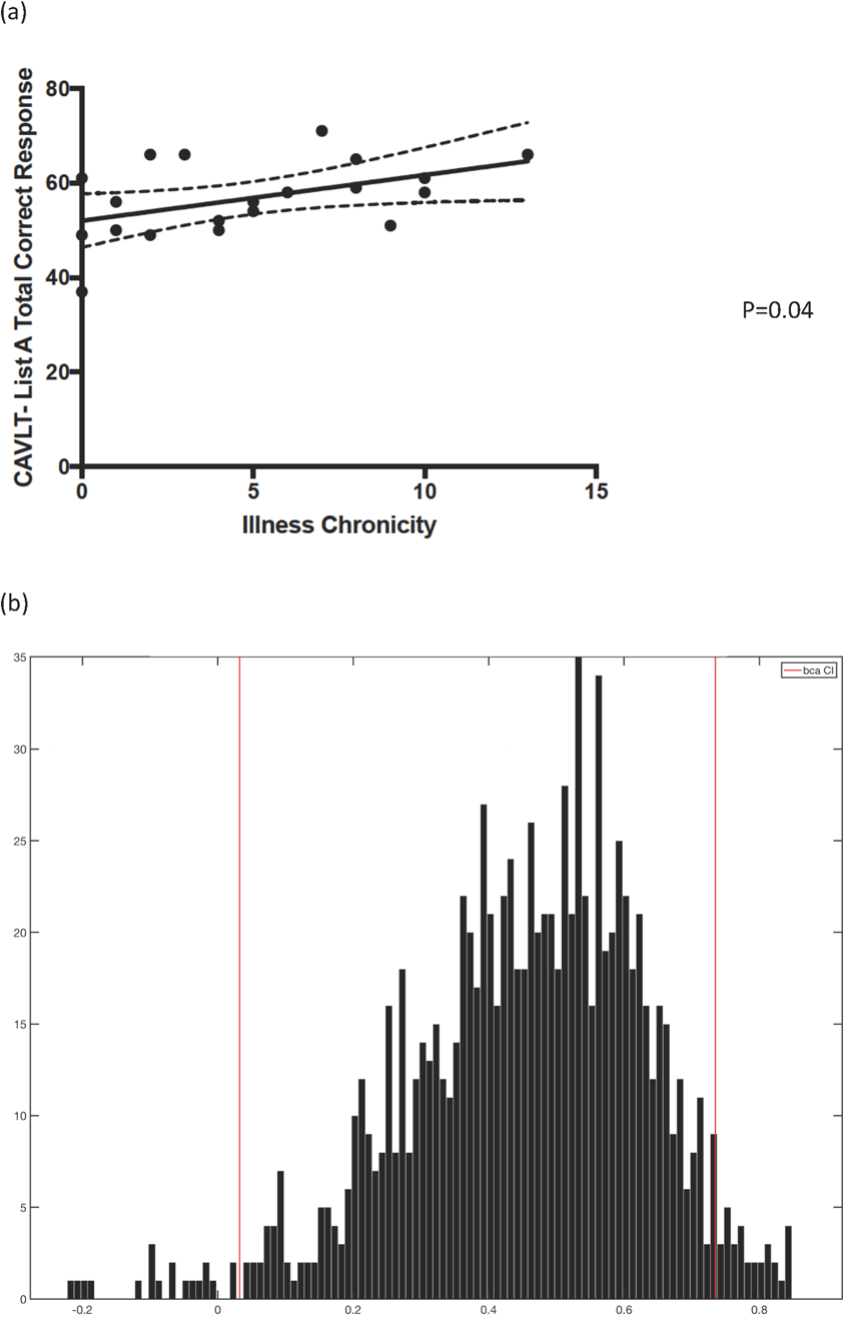
Illness chronicity (defined as years since first depressive onset) significantly correlated with verbal memory (CAVLT List A total). 95% C.I. of Bootstrapped correlation coefficients: 0.03–0.74. *CAVLT = Chinese Auditory Verbal Learning Test*. (**a**) Correlation between Illness Chronicity and CAVLT List A Total Correct Response score.(**b**) Histogram showing bootsttrapped correlation coefficients between Illness chronicity (years since depressive onset) and CAVLT- List A Total Correct Responses.

**Correlation is significant at the 0.01 level (2-tailed). *Correlation is significant at the 0.05 level (2-tailed).

(Figure 1) Illness chronicity (defined as years since first depressive onset) significantly correlated with verbal memory (CAVLT List A total). Lifetime number of hypomanic or depressive episodes did not correlate with any cognitive variables. The correlation of visual memory (immediate recall) with anxiety became insignificant after bootstrapping.

### Cognitive correlates –Unipolar Depressed Subjects

**(**Table 5**)** In UD subjects, no correlation was found between cognition and any course variables. Depressive severity did not correlate with TMT-A, TMT-B or any other cognitive variables. The correlation of verbal fluency-correct score was rendered non-significant after bootstrapping.

**Table 5:** Pearson correlation coefficients (Bootstrap BCa 95% confidence intervals) of correlations of cognitive test (ES > 0.30 versus HC) with symptomatic and course variables in Unipolar Depressed subjects.

Unipolar Depressed subjects N = 35	MADRS	HAM-A	Illness Chronicity	Number of depressive episodes	Cycling frequency
**Frontal Executive Function**					
WCST % perseverative error	−0.06 (−0.35–0.25)	−0.10 (−0.46–0.49)	0.00 (−0.31–0.45)	−0.02 (−0.31–0.35)	−0.10 (−0.33–0.18)
Categories completed	−0.25 (−0.59–0.06)	−0.08 (−0.54–0.40)	0.21 (0.05–0.35)	−0.07 (−0.52–0.31)	−0.25 (−0.75–0.13)
**Attention and mental tracking**					
TMT-A	0.14 (−0.16–0.42)	0.11 (−0.26–0.40)	0.16 (−0.07–0.40)	0.10 (−0.19–0.41)	0.15 (−0.01–0.38)
TMT-B	0.001 (−0.36–0.43)	0.12 (−0.23–0.47)	0.19 (−0.18–0.51)	0.05 (−0.30–0.45)	−0.05 (−0.16–0.05)
**Verbal Fluency**					
Correct	−0.04 (−0.46–0.43)	0.35* (−0.06–0.69)	0.10 (−0.21–0.40)	−0.23 (−0.52–0.17)	−0.33 (−0.58–0.02)
**Learning and memory**					
Wechsler Memory Scale (Visual Reproduction)					
Immediate recall	0.13 (−0.14–0.36)	−0.06 (−0.39–0.29)	−0.07 (−0.45–0.31)	−0.03 (−0.35–0.31)	0.10 (−0.11–0.33)
Delayed recall	−0.13 (−0.36–0.11)	−0.06 (−0.41–0.28)	−0.14 (−0.41–0.12)	−0.06 (−0.41–0.28)	0.12 (−0.13–0.42)

*Correlation is significant at the 0.05 level (2-tailed).

## Discussion

To our knowledge, this is the first study examining cognitive functions of bipolar II and unipolar depressed individuals with no prior psychotropic treatment. Our subjects were young, currently moderately depressed and on average within 3–5 years from first depressive onset, with no history of psychosis or psychoactive substance use.

Our main finding was that treatment-naïve young individuals with BPII had cognitive function distinguished from unipolar depressed individuals by an intact cognitive speed (TMT-A completion). UD patients had more significant impairment in executive function. In particular, cognitive function (verbal memory) correlated with illness chronicity only in bipolar II but not unipolar depression. The findings were interesting in a number of ways.

Firstly, the relatively intact cognitive function in our depressed BPII patients was consistent with those reported by Taylor Tavares *et al*.^14^, which compared unmedicated bipolar II with unipolar depressed patients where unipolar patients showed an increased level of cognitive impairment. It is likely that the discrepancy from reports of impaired attention and memory in medicated bipolar II subjects^15–17^ was attributable to the effect of medications, as all studies reporting marked cognitive impairment in Bipolar II involved medicated samples, whereas the unmedicated patients in Taylor Tavares *et al*. had on average 15–18 years of illness too.

Secondly, that UD subjects had slower cognitive speed (TMT-A completion time) than BPII depressed subjects was consistent with clinical observation that psychomotor retardation tended to characterize UD as opposed to psychomotor activation in BPII depression^18^. The more prominent impairment of cognitive flexibility in UD instead of BPII compared to healthy controls was also compatible with the findings of Taylor Tavares *et al*.^14^, but larger samples may help clarify the trend-level increase observed here.

These may also suggest fundamentally distinct neuroanatomical and functional changes observed in UD and BPII depression. Cognitive processing speed has been attributed to global white matter volume, structural integrity of white matter tracts in bilateral parietal and temporal lobes, along with left middle frontal gyrus, which correspond to the trajectories of the superior and inferior longitudinal fasciculi^19,20^. While there is a lack of neuroimaging data on treatment-naïve bipolar subjects, an intact neurocognitive performance in BPII contradicted with structural imaging studies which demonstrated increased deep white matter hyperintensities (DMWH) in bipolar I versus unipolar depressed patients, particularly the left superior longitudinal fasciculus^21^. It is however likely that broader white matter deficits would appear only later in the course of illness as a consequence of energy metabolism disturbances in bipolar disorders^22^. Prospective investigation of white-matter changes and their correlation with processing speed in bipolar II subjects would be needed to clarify this issue.

Thirdly, our exploratory analysis interestingly showed correlation between disease chronicity and verbal memory (CAVLT List A Total) only in BPII but not UD. The different implications of verbal memory deficit in UD and BPII is compatible with the existing literature. A recent meta-analysis found verbal memory in patients with Major Depressive Disorder to be correlated with current symptoms while attention and executive dysfunction to be compatible with trait deficits^23^. Previous studies also found verbal memory impairment in Bipolar I and II to be a stable deficit, reflecting illness chronicity and a trait marker for manic episodes^3,24–27^. Although we did not observe significant between-group differences in verbal memory, possibly limited by sample size, the chronicity correlation in bipolar II appeared compatible with existing literature in being a phenomenon specific to bipolarity.

There are a few limitations in our study. First, in spite of the difficulty in recruiting untreated bipolar II and unipolar depressed patients of comparable severity, the modest sample size (20 BPII, 35 UD, 35 controls) meant that there was only sufficient power to detect a between-group difference of large effect size = 0.80 or above between the 20 BPII and 35 UD or HC subjects, and in the BPII subjects, there was sufficient power to detect correlations at effect size of at least 0.55. For example, there was insufficient statistical power (0.49) to detect significant difference in TMT-B, of effect size = 0.42 that was found between the BPII and UD subjects. Second, the cross-sectional nature of our study may limit precision of course variables such as age of onset, frequency and duration of past affective episodes, and preclude examination of causality. Prospective longitudinal studies may minimize these biases but the effect of medications would also become difficult to eliminate. Third, we did not include bipolar I depressed individuals in the study which would otherwise even more clearly establish the value of cognitive differences in bipolar/unipolar distinction. Given the difficulty in recruiting individuals with bipolar I disorders in an unmedicated state – as they would more likely stay on or were given mood stabilisers given the disruptive nature of their manic states, such effort should be considered in the context of a multi-centre study. Lastly, given that this study only involved young, unmedicated and currently depressed patients, there remained a likelihood that some of these currently depressed patients with no overt hypomanic features will eventually develop hypomanic episodes. This may have resulted in an underestimate of trait differences between the bipolar and unipolar groups. Inclusion of subjects with longer durations of affective illness would give better assurance of diagnostic stability of the unipolar depressed group identified, while a longitudinal study to follow-up on the cognitive and symptomatic profiles of these patients would allow re-analysing the baseline data in light of updated clinical categorisation upon follow-up, but also help identify baseline predictors for later conversion to bipolarity.

In conclusion, we found young and treatment-naïve patients with BPII depression to be distinguished from unipolar depressed patients by a relatively intact cognitive profile, with a verbal memory-chronicity correlation that suggested a stronger resemblance of BPII to BPI rather than UD. Our data did not provide direct support to the hypothesis of neuro-progression, although the relative proximity of our subjects to illness onset did sensitise our data to trait correlates rather than neuroprogressive consequences. Longitudinal studies of longer follow-up duration on cognition of Bipolar II individuals is warranted to examine for any illness progression effects. The contrast of our findings to those from medicated samples also indicates need for closer examination on the cognitive effects of medications used for treatment of patients with bipolar disorders, irrespective of efficacy on affective symptoms.

## Methods

### Participants and Recruitment

Given the scarcity of drug-naïve patients, we comprehensively recruited all treatment-naïve, currently depressed patients aged 18 to 35 presenting to a specialist psychiatric clinic in the years 2014–2017. Inclusion criteria were as follows: (i) age 18 to 35, (ii) currently satisfying the criteria for DSM-IV-TR Major Depressive Episode, (iii) Unipolar depression as defined by meeting DSM-IV-TR criteria for Major Depressive Disorder, with no history of hypomanic episodes, Bipolar II disorder as defined by RDC criteria (DSM-IV-TR major depressive episode with history of hypomanic episodes of at least 2-day duration)^28^; (iii) and had no prior exposure to any psychotropic drug treatment in their lifetime. All research assessments were made before the first clinic appointment. Diagnostic assessments were conducted by trained interviewers using the Chinese bilingual version of the SCID-I adapted to facilitate diagnosis of current and lifetime hypomanic episodes under the supervision of an experienced clinician academic psychiatrist^8,28,29^. All lifetime affective episodes were enquired year by year from the first onset of depression using a modified life-chart method based on SCID-I. Repeated interviews with patients and informants were sought to confirm that past hypomanic episodes were observable. Exclusion criteria included current and lifetime histories of psychoses, substance misuse, organic brain syndromes, or evidence of intellectual disability. Healthy volunteers with no personal or family history of any mental disorders were recruited from online advertisements.

Matching of healthy controls with the UD and BPII groups was done on a group level so as to constitute similar age, gender and education in healthy controls compared to the subject groups combined. We were unable to conduct age/gender/education matching between the UD and BPII group owing to the scarcity of treatment-naïve and currently depressed subjects, especially those with BPII, meeting the recruitment criteria.

All eligible participants provided valid written informed consent. All experimental protocols were approved by the New Territories East Cluster- Chinese University of Hong Kong Clinical Research Ethics Committee and all procedures were performed in accordance with the approved guidelines and regulations.

### Cognitive and Clinical Assessments

Current-week affective symptoms were evaluated by trained clinician interviewers with the interviewer-administered Montgomery-Åsberg Depression Rating Scale^30^, Young Mania Rating Scale^31^, and Hamilton Anxiety Rating Scale^32^. Current health-related quality of life was rated with the Short-form-36 health survey (SF-36)^33^, a popular generic self-rated measure of health-related quality of life, validated for Chinese settings^34^. Medication history was confirmed, apart from direct enquiry with the subjects and caregivers with a multi-tiered approach – (i) current and lifetime use, adherence, and dosages of all psychotropics were retrieved from a territory-wide hospital computerized management system (ii) Access to family physician or private practitioners with the subjects’ consent (iii) Pill count.

A battery of neuropsychological tests was used to assess a broad range of cognitive functions:

Attention and mental tracking-

 Processing Speed - Trail-making test A^35^

 Attention - Digit Span Forward, Digit Span Backwards^36^,

 Attention switching - Trail-making Test B^35^

Frontal Executive Function-

 Wisconsin Cart Sorting Test^37^

 Verbal fluency – Category fluency Test^38^

 Verbal Memory – Chinese Auditory Verbal Learning Test^39^

 Visual Learning – Wechsler Memory Scale- Visual reproduction^40^

The Three-subtest Short Form of the Wechsler Adult Intelligence Scale-III was conducted in all participants to assess general intelligence^40,41^.

### Statistical Analysis

SPSS v24.0 was used for data analysis. The demographic characteristics and diagnostic variables are assessed using Chi-square for categorical variables, unpaired t-test for continuous variables. Scaled neuropsychological test scores, MADRS, HAMA, YMRS and SF-36 scores were compared between groups with one-way ANOVA. Post-hoc Bonferroni’s correction for multiple comparisons was used in between-group pairwise comparisons. Welch’s F statistic was used for comparison of continuous variables where variances were not equal between groups. Pearson’s correlation was used for exploratory analysis of correlation between mood ratings, course variables and neuropsychological test scaled scores on tests that showed at least a small effect-size (Cohen’s d =/> 0.2) compared to healthy controls. Bootstrapping was applied for the correlational analysis to adjust for effect of outliers. A two-sided P-value of less than 0.05 is considered significant. Given its exploratory nature, we did not apply corrections for multiple comparisons in the correlational analyses, but only correlations of p value less than 0.05, and with BCa 95% C.I. not including zero would be considered significant.

### Data Availability Statement

The datasets generated during and/or analysed during the current study are available from the corresponding author on reasonable request.
